# Introducing the Democratic Electoral Systems data, 1919-1945

**DOI:** 10.12688/openreseurope.17264.2

**Published:** 2024-09-06

**Authors:** Nils-Christian Bormann, Lea Kaftan

**Affiliations:** 1 Department of Philosophy, Politics & Economics, Witten/Herdecke University, Witten, North Rhine-Westphalia, 58455, Germany; 2 GESIS Leibniz Institute for the Social Sciences, Cologne, North Rhine-Westphalia, 50667, Germany

**Keywords:** electoral rules, parliamentary elections, presidential elections, democracy, proportional representation, majoritarian systems

## Abstract

This data note introduces an update to the widely-used Democratic Electoral Systems (DES) data that encompasses the period from 1919 to 1945. The data include 243 legislative lower house and presidential elections in 34 interwar democracies. Information on these elections falls into four categories: first and foremost, DES contains variables that capture the institutional rules that define how elections are organized. Second, the data captures the consequences of electoral rules in the form of summary statistics of electoral outcomes. Third, we include democracy classifications for four major democracy datasets so that users can choose their preferred democracy definition when working with the data. Finally, the DES dataset contains multiple identification variables that allow linking the DES data to a wide variety of other datasets. This update to the DES data is fully compatible with prior releases for the post-war period ^1– 3^.

## Plain language summary

We provide information on electoral rules between 1919 and 1945. We focus on parliamentary and presidential elections in democracies. We collect information on these rules in a dataset of 243 elections. Many rules exist. Some describe how many members a parliament has. Others determine where politicians are elected. Again others decide how individual votes a party needs to get a seat. We also describe the consequences of elections, for example, how many parties make it into parliament. There are different ways to think about what democracy is. Our dataset uses four different approaches to determine whether or not a country is a democracy so users can decide which elections to consider democratic. Our data can also be linked to many other data sources.

## Introduction

We are introducing an update to the widely-used Democratic Electoral Systems (DES) data that encompasses the period from 1919 to 1945. The data include 243 legislative and presidential elections in 34 interwar democracies. Information on these elections falls into four categories: first and foremost, the DES data contains variables that capture the institutional rules that define how elections are organized. Second, the data captures the consequences of electoral rules in the form of summary statistics of electoral outcomes. Third, we include democracy classifications for four major democracy datasets so that users can easily choose their preferred democracy definition when working with the data. Finally, the DES dataset contains multiple identification variables that allow linking the DES data to a wide variety of other datasets.

In addition to a new variable on the constitutionally prescribed length of time between elections, this release of the historical DES data contains all variables of prior releases for the post-war period, and is thus fully compatible with them ^1– 3^. These DES versions have been used to analyse a broad range of questions in political science, economics, sociology, and related disciplines. Most recently, social scientists employed the data to explore outcomes such as affective polarization ^4^, voting preferences in general ^5^, for left and right-wing parties in particular ^6^, and public participation in policy-making ^7^. Prior versions of the data on electoral rules entered analyses of party systems ^8^, party breakdown ^9^, foreign direct investment ^10^, ethnic coalitions ^11^, the development of legislatures in Africa ^12^, and elite reactions to far right challengers ^13^. All these studies are highly pertinent to studying the future of democracy. ^1^

Although this release of the DES data covers a historical period, studies of the tumultuous interwar years might be able to teach us something about the fate of democracies today ^14^. Studying electoral choices and reforms during the interwar period can inform debates about contemporary institutional changes. Historical party systems are relevant comparison cases for studies of the effects of party fragmentation today ^15, 16^. Finally, the DES 1919–1945 release will prove a valuable resource for scholars who study the origins of electoral systems, for example, to explore the diffusion of electoral rules across Europe and the Americas ^17^.

In the following, we describe how we collected the data, how we ensure data quality, and provide a range of summary statistics that describe the data.

## Materials & methods

The DES data assembles and harmonizes previously scattered and analogue sources into one data set ^18^. Whenever possible, we rely on primary sources, such as official election returns from statistical or government agencies, and electoral laws. Edited volumes that unite case studies on electoral systems and electoral returns constitute crucial secondary sources ^19– 22^. We cross-referenced different sources to ensure data validity. When sources disagreed, we provide the information given by a majority of sources or contacted country experts.

Data collection relies on the experiences made in three previous rounds of post-World War II DES releases ^1– 3^ and proceeded in six steps:

1. We defined the sample of elections to be included in the data. All elections must be democratic according to one of four major democracy indices: the dichotomous measure by Boix, Miller & Rosato ^23^, the dichotomous *Democracy and Dictatorship* (DD) classification ^24, 25^, the ordinal *Polity5* index ^26^, and the continuous *Varieties of Democracy* (V-Dem) Polyarchy scale ^27^. Since the original DD classification only starts in 1946, we classified all elections in the data according to its rules.
2. We trained student research assistants (RAs) on the coding rules of the DES data ^1, 3^. The training involved a theoretical overview of electoral rules, the introduction of relevant source material, and example classifications of two elections.
3. Each RA classified the same set of ten randomly selected elections. We compared RA classifications to our own classification of these elections, and provided individual performance feedback to each RA.
4. We divided the election sample between RAs according to linguistic expertise. Between ourselves and the RAs, we were able to read source material in Dutch, English, Finnish, French, German, Italian, Serbo-Croatian, Spanish, and Swedish. In total, we spoke the languages used in 26 out of the 34 countries included in the dataset. For the remaining eight countries, we relied on secondary sources or contacted country experts. We encouraged RAs to contact us with questions when they were uncertain how to classify a particular variable or election. When our reading of sources could not provide an answer to unclear cases, we contacted other leading experts on electoral systems and/or particular countries to help us reach a decision.
5. We randomly re-sampled about 10% of elections and reclassified them to see if systematic errors occurred, and corrected them where necessary.
6. Specific variables are automatically created. For example, we compute the effective number of electoral parties ( ENEP) at each election through an algorithm that summarises election results into the overall index. For most elections, we link DES elections to electoral results collected by the authors in a different dataset, the *Actions by Elites and Leaders* (ABEL) data ^16^. For all other elections, we collected raw electoral results from the sources described above.
7. In a final step, we ran an automated script across the entire data to check each variable for its consistency with the coding rules, which identifies typos and other data entry mistakes. The script also ensures inter-variable consistency and thereby guarantees that two variable values do not contradict each other. For example, if the variable legislative_type indicates the use of proportional representation (PR) in a particular election, we checked that the variables elecrule and tier1_formula indicate specific PR sub-types but not sub-types of any other electoral family.

The dataset comes with several identification variables that make it interoperational with other scientific and public-use datasets. Most users of the DES data are likely to link it to other country-level datasets. Thus, next to a unique variable that identifies each election ( elec_id), the dataset provides several country-identifiers, including the country name, the country abbreviation, the Alvarez, Limongi, Cheibug & Przeworski country codes ( aclp_code) ^24^ along with the widely-used Correlates of War (COW) country codes ( ccode) ^28^ and the Gleditsch & Ward (GW) country IDs ( ccode2) ^29^. Unlike identification systems of country names and different ISO country abbreviations, the COW and GW identification variables accurately trace the historical development of the international system. ^2^

## Democratic Electoral Systems, 1919–1945

To accommodate different views of democracy, we identify democratic elections according to four different democracy definitions. Each election in the DES 1919–1945 has been classified as democratic by at least one of four datasets. Two inclusive classifications identify almost all 243 elections in the dataset as democratic. Boix, Miller & Rosato’s (BMR) Complete Data of Political Regimes and Pzeworski et al.’s Democracy and Dictatorship (DD) data. The former consider all but two Spanish parliamentary elections in 1919 and 1920 as democratic ^23^. DD neither classifies the two Spanish elections as democratic, nor three elections in San Marino ^24^. ^3^ The more complex V-Dem and Polity5 indices categorize 185 and 173 elections respectively as democratic. Users of our data thus have the choice to pick their preferred definition of democracy and the associated sample of elections, or to take an inclusive approach by picking all elections that have been classified as democratic by at least one democracy indicator. In the following, we present descriptive statistics for all election recognized as democratic by at least one of the four democracy indicators.

**Figure 1.**
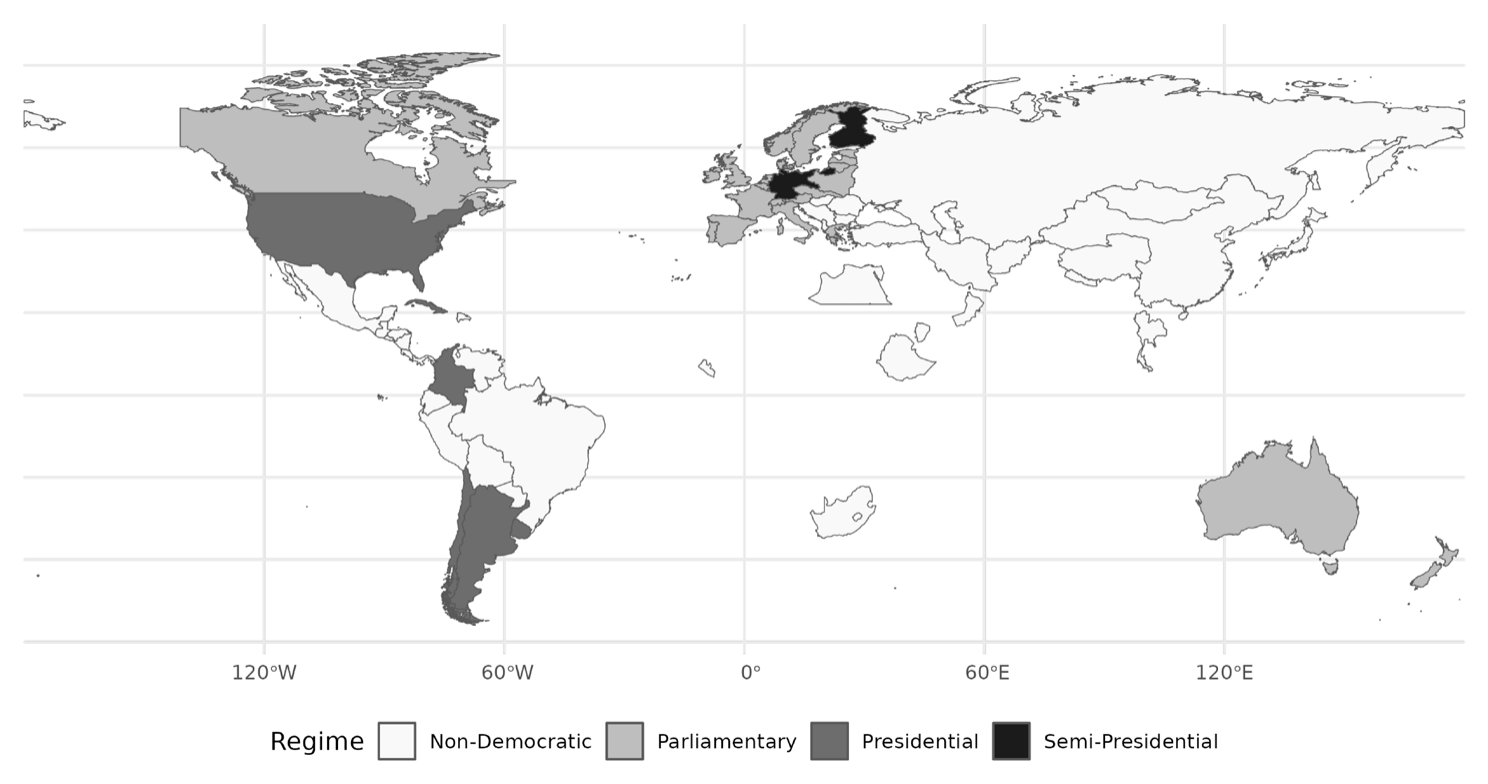
Regime types across the world. First election per country depicted.

Our data includes information on 213 legislative, lower-house and 30 presidential elections in 34 democracies between January 1st, 1919 and December 31st, 1945. Figure 1 shows the distribution of regime types across the globe. 26 states featured a parliamentary regime (light grey), two a semi-presidential form of government (black), and four were presidential (dark grey). ^4^ Geographically, the majority of democratic states were situated in Europe (25), while the rest clustered in Latin America and the Caribbean (5), in North America (2), and in the Pacific (2). ^5^

**Figure 2.**
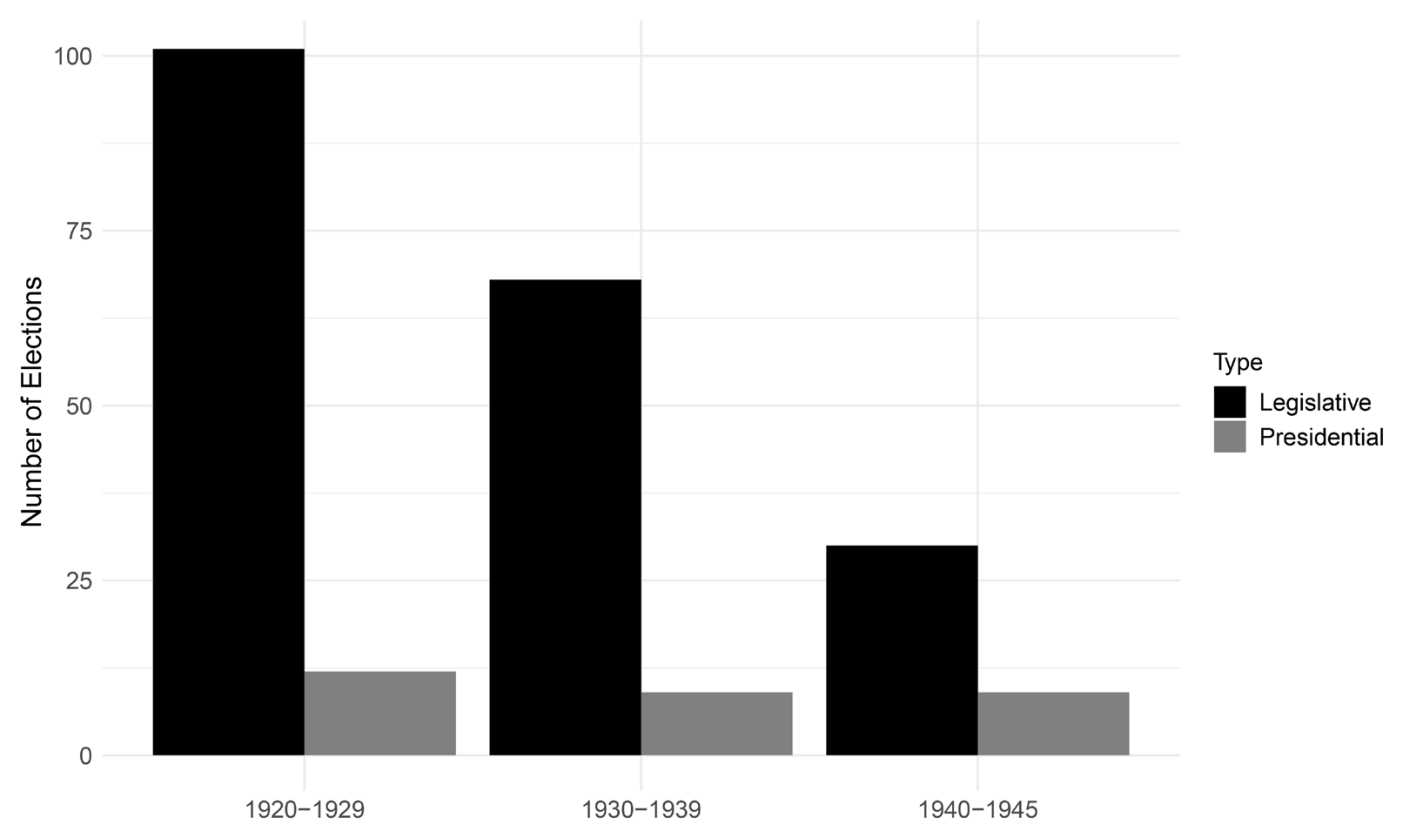
Democratic elections per decade, 1920–1945.

Figure 2 depicts the frequency of legislative and presidential elections per decade. ^6^ The declining number of elections tracks the breakdown of democracy in many European states. Notably, the steeper decline in legislative compared to presidential elections contrasts with the greater instability of presidential systems in the post-World War II period ^30^. This observation raises theoretical questions regarding the stability of presidential versus parliamentary systems, which we would not be able to investigate further without data from the interwar period.

To enable users to compare the frequency of elections between different democracies, we added a new variable that describes the constitutionally prescribed duration in years between legislative and presidential elections. For example, members of the United States' House of Representatives run for office every two years, while parliamentarians in interwar Czechoslovakia only faced voters every six years. We record the longest electoral cycle in our data for presidential elections in Germany, which took place every seven years. Moreover, we also collected information on the partial renewal of parliament. For example, lower house elections in Argentina take place every two years for 50% of MPs. These on term lengths do not only allow users to compare another characteristic of electoral rules but also facilitate weighting the number of units per country in cross-country quantitative comparisons. We publish them in a separate data sheet to ease merging the interwar data with its post-World War II counterparts.

### Legislative elections

**Figure 3.**
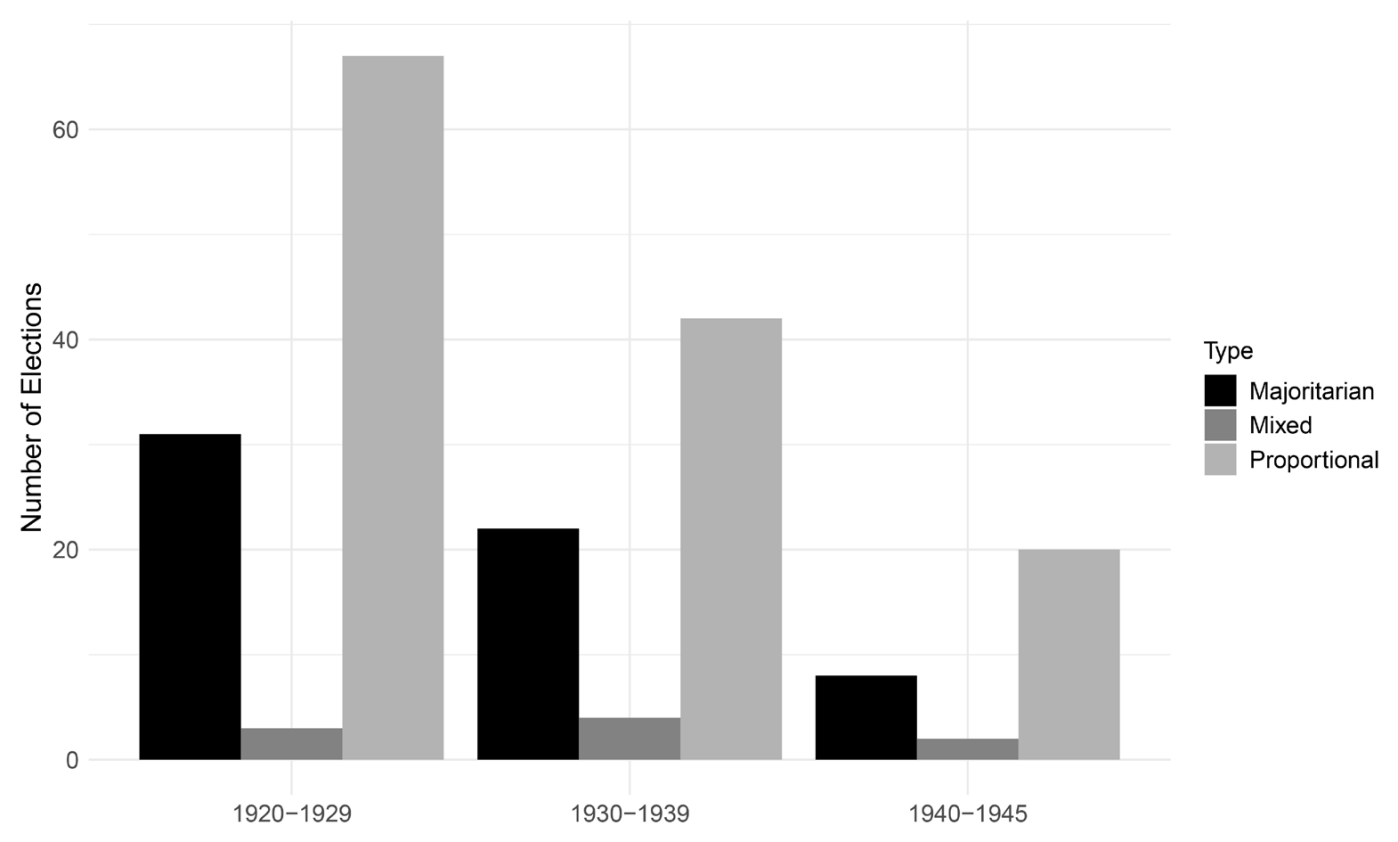
Legislative families per decade, 1920–1945.

Most of the information in the DES data focuses on legislative elections. The included variables provide different classifications of electoral families, electoral rules, their application and combination within or across different levels of aggregation or electoral tiers, the number and average size of districts, and the resulting number of parties at each election. Figure 3 displays the distribution of legislative electoral families per decade. During the interwar period, electoral rules that translate votes proportionally into seats were roughly twice as common as majoritarian systems which award seats to the candidate(s) with the highest vote total(s) in a district. Combinations of proportional and majoritarian rules, so-called mixed systems, were uncommon during the interwar period, and only used in France and Iceland.

**Figure 4.**
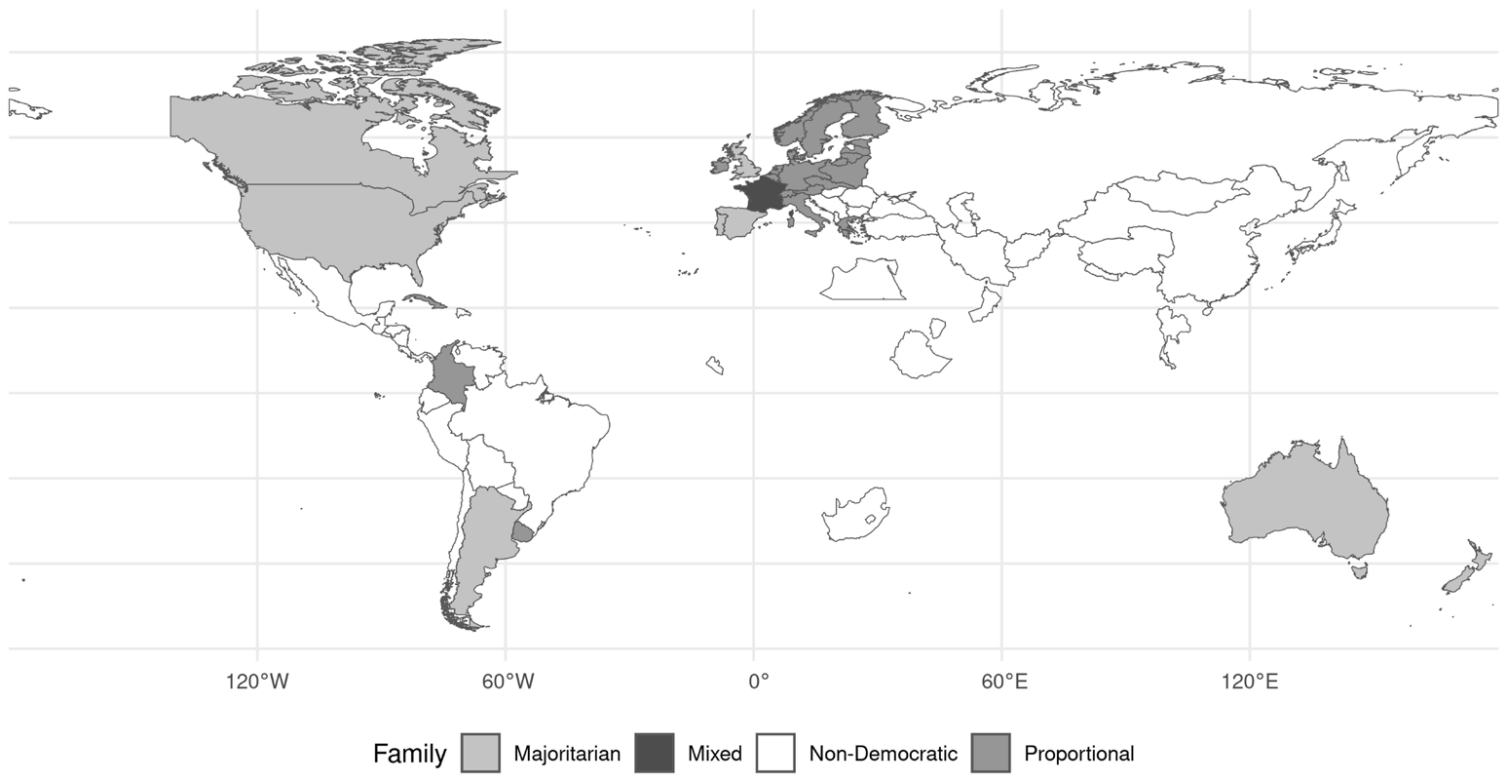
Legislative types across the world. First election per country depicted

Figure 4 shows that list PR systems were the most popular proportional rule with the D'Hondt divisor as the most common way to allocate votes into seats (49\% of all PR elections). The remaining PR elections employed various quota systems, such as the Hare (17.27\%), Hagenbach-Bischoff (15.83\%), or Droop quota (7.19\%) (see Figure 4). Single-member district pluralities or first-past-the-post elections constituted the preferred choice within the majoritarian electoral family (54.69\%).

Geographically, all Anglo-Saxon states ran their elections under majoritarian rules as Figure 4 shows. Spain, during its first democratic spell in the early 1920s, also operated a first-past-the-post system. The other two majoritarian states, Argentina and Portugal, used the limited vote---an electoral system with multi-member districts, in which voters have fewer votes than there are seats. Besides the majoritarian United Kingdom and France, all other European countries had adopted PR in the early 1920s. Among the PR states, only Ireland departed from the list-PR consensus, and used the Single Transferable Vote (STV) instead. France was the only continental European state that held two of its five elections under mixed electoral rules. The only other mixed electoral system in our sample was employed in Iceland. During the interwar period, the country was part of a union with Denmark and not yet internationally recognized, which is why it is not depicted in Figure 4. Its voters elected four MPs by D'Hondt in the Reijkjavik constituency in addition to 12 seats in six two-member districts and 20 seats in single-member constituencies by plurality. In the 1919 and 1924 elections, France employed a mixed fusion system in multi-member districts. Voters had as many votes as there were seats in a given district (Borda Count) and candidates were elected if they received more votes than there were voters in a given district. Candidates were also part of lists. In a second stage, list votes were distributed by the Hare formula. If turnout in a constituency was lower than 50% or if no list passed the electoral quota a second round was to take place 14 days later in which a relative majority of votes was sufficient.

Two democracies used electoral systems that have not been used by any other state in national elections during the time period covered by any previous DES data release (1946–2020), and thus enter the DES data for the first time. Germany used the *fixed quota* system that distributed one seat for 60,000 votes ^31,73–81^. Unlike most quota systems, which fix the number of parliamentary seats and determine the quota after counting the votes, the fixed quota system reverts this relationship. It sets the quota first and then determines the number of parliamentary seats. This practise led to an ever larger parliament as Germany’s population and electoral participation grew. The size of the German Reichstag swelled from 459 deputies in 1920 to 647 members after the final election in March 1933. The second unique system, the *cumulative vote*, was employed in Chile until 1921 ^32^. It is similar in all but one respect to the majoritarian *block vote* system, in which voters have as many votes as there are seats in multi-member districts. However, voters can award more than one vote to a candidate under the cumulative vote.

**Figure 5.**
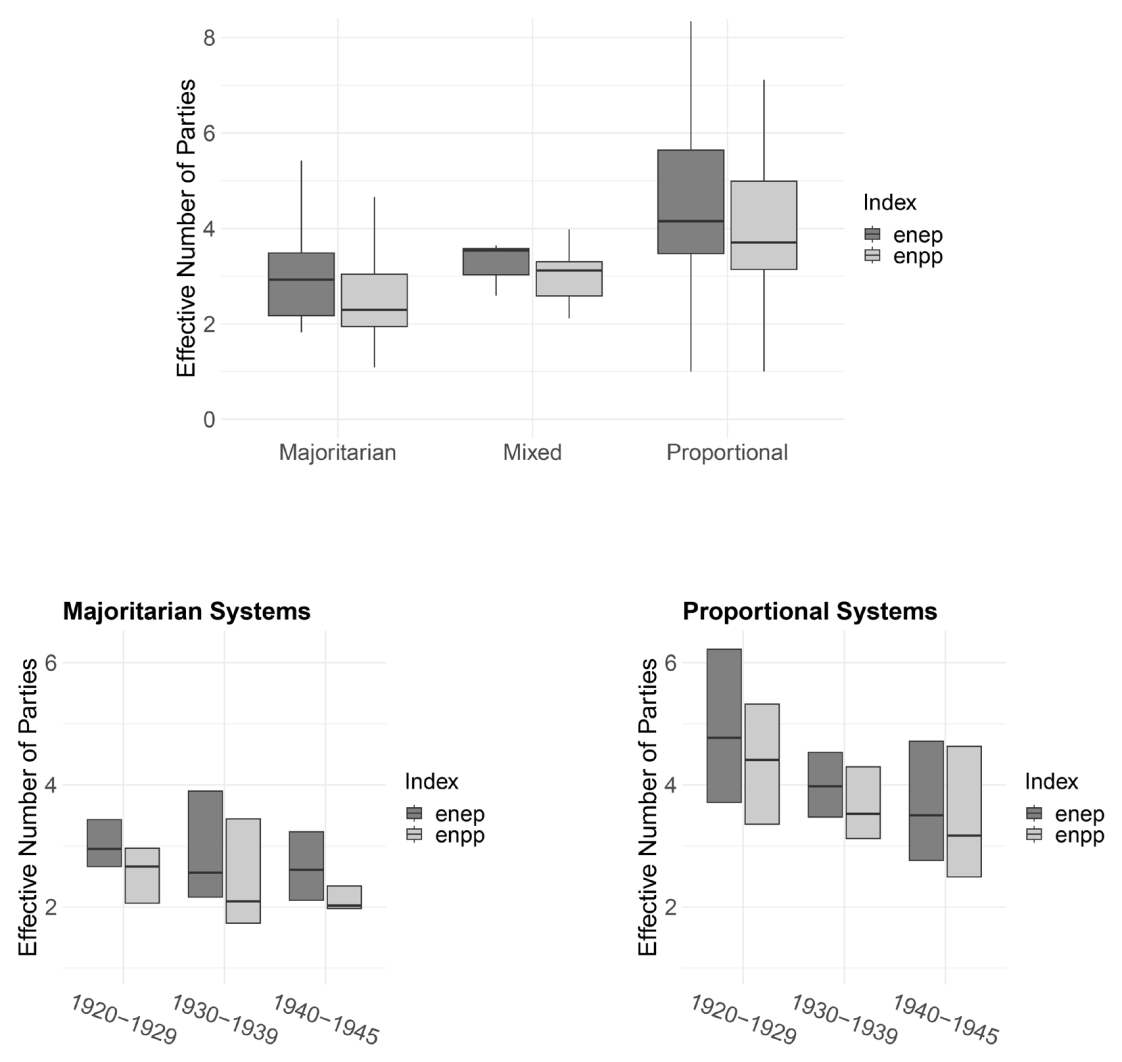
Party system size by electoral system family, 1919–1945.

Next to detailed information on legislative electoral rules, the DES data also provide insight into the consequences of these rules in the form of party system size figures. Figure 5 presents box plots of the effective number of electoral ( enep) and parliamentary parties ( enpp) across electoral families (top) and within familes over time (bottom). ^7^ In line with theoretical predictions ^33^, majoritarian systems are associated with the smallest number of parties while PR systems are most permissive. From 1920 to 1940, the effective number of parties in PR systems decreases more steeply than in majoritarian systems (bottom panels). In part, this may be a selection effect where countries with more parties where more likely to fail. For example, four of the five democracies with the highest ( enep) scores failed in the interwar period. ^8^

### Presidential elections

Presidential elections constitute just over 12% of all elections in the DES, 1919–1945 data. As shown in Figure 1 presidential regimes clustered in the Americas. European democracies were overwhelmingly of parliamentary types with semi-presidential exceptions in Finland, Germany, and Ireland after 1937. This low share contrasts markedly with the post-World War II period, when more than a quarter of all elections were presidential ^1, 3^.

**Figure 6.**
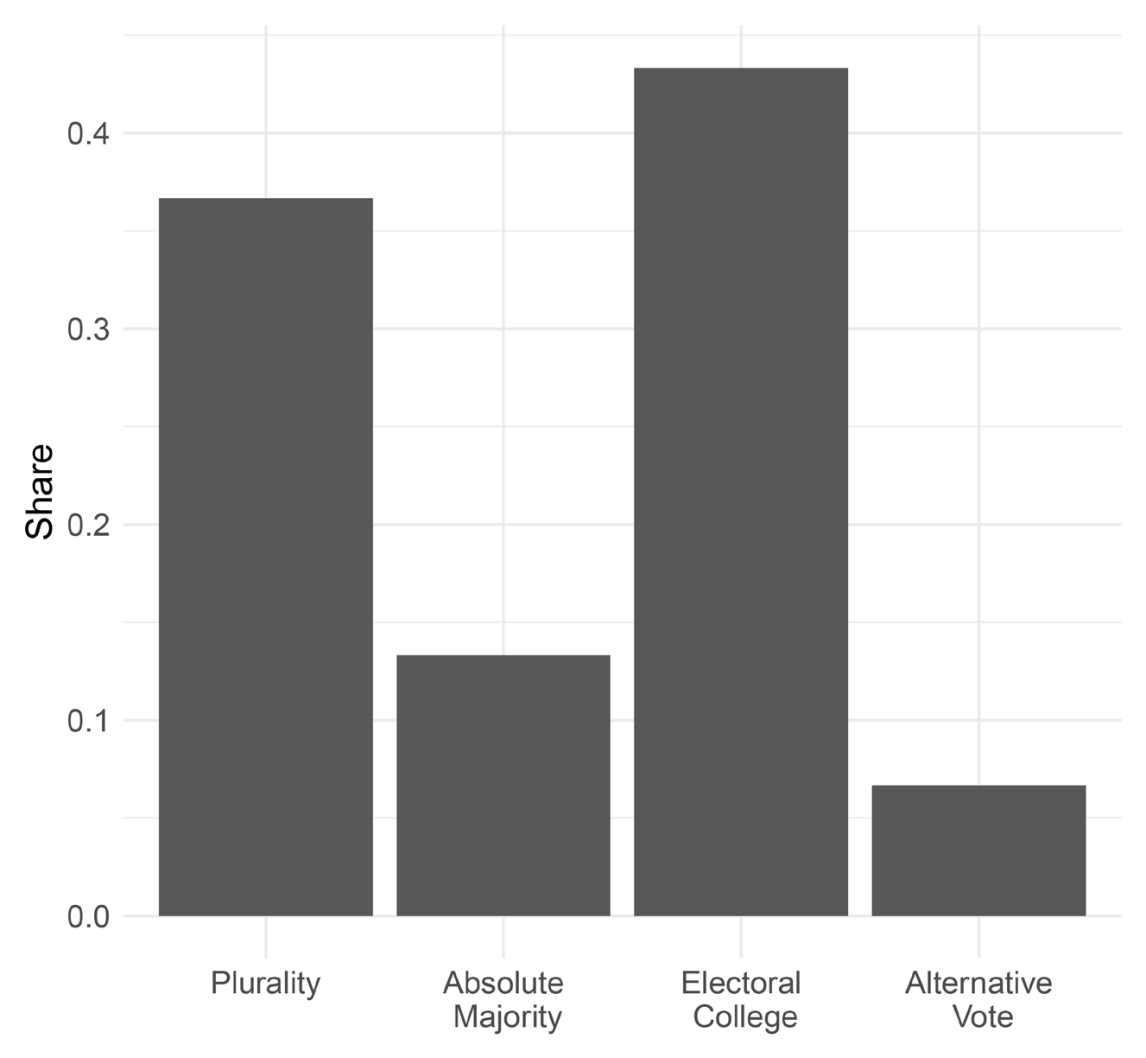
Presidential electoral rules, 1919–1945.

Mirroring the early post-World War II decades, the electoral college was the most common electoral system used in presidential elections during the interwar period, closely followed by plurality elections (see Figure 6). Absolute majority systems and the alternative vote, that was only used in Ireland, were employed in less than 20% of all presidential elections.

Finally, we do not find any notable association between presidential electoral rules and the number of candidates. Whereas plurality systems are associated with a smaller and less variable number of presidential candidates relative to absolute majority systems in the 20th and 21st centuries ^3, 34, 35^, we find virtually no differences between electoral rules in the period 1919–1945. This null finding may in part be explained by the small number of elections observed during the period that does not result in sufficient variation. Another factor could be the relatively young age of interwar democracies. Out of ten states that held presidential elections, only the United States had been a democracy for more than a decade before 1919, when our dataset starts recording elections. Candidates and voters might have had too little time to learn about the mechanical and strategic effects of electoral rules. This difference in the associations between different features of electoral systems for periods before and after 1945 is of theoretical relevance. We want to encourage researchers to explorer these differences further. 

## Conclusion

In this data note, we described an update to the Democratic Electoral Systems (DES) data that extends the coverage to the period 1919–1945. Recently, concerns about the quality and survival of contemporary European and other long-established democracies are on the rise. Our new data can help answer questions about the association of parliamentary and presidential institutions as well as party system fragmentation on one hand, and the fate of democracies in another troubled period on the other. The DES 1919–1945 data might also be of use to scholars interested in the origins of electoral rules or long-term institutional legacies.

## Data Availability

All figures and reported statistics can be replicated with R scripts contained in the dataverse. The authors ran the scripts on R version 4.3.1 (“Beagle Scouts”) and used the R packages *colorspace* ^36^, *cshapes* ^37^, and *ggplot2* ^38^. Harvard Dataverse: Replication Data for: Introducing the Democratic Electoral Systems data, 1919–1945. https://doi.org/10.7910/DVN/FWLXHM ^18^ This project contains the following underlying data: Electoral rules data: “es-1919-1945-231204.csv” Electoral period data: ``es-1919-1945-term-lengths-240826.csv'' Electoral results data: “es-1919-1945-results-231204.csv” Codebook: “es-1919-1945_codebook.pdf” Summary file: “readme.txt” Data are available under the terms of the Creative Commons Zero "No rights reserved" data waiver (CC0 1.0 Public domain dedication). To replicate the figures in this study unpack the zip archive and R replication file: “data-es_interwar-master-231204.R” R replication file for Figure 1: “des_figure_regimetype.R” R replication file for Figure 2: “des_figure_elecdec.R” R replication file for Figure 3: “des_figure_elecfamdec.R” R replication file for Figure 4: “des_figure_elecfam_map.R” R replication file for Figures. 5, 5a & 5b: “des_figure_enep_enpp.R” R replication file for Figure 6: “des_figure_preselecrule.R”

## Footnotes

^1^Overall, the the dataset has been cited 1439 times according to a Google Scholar query on 26 August 2023.

^2^The two slightly differ in how they identify ancestor and successor states. For example, the COW system sees unified Germany after 1990 as a successor to Imperial/Nazi Germany before 1945, whereas GW classify unified Germany as a successor of West Germany.

^3^Both BMR and DD do not consider the Spanish elections as democratic because the unelected Spanish monarch could dismiss governments and revoke laws. The DD rules further dismiss the San Marino elections because the country did not experience an alternation in executive power in the period 1906–1922.

^4^Parliamentary regimes feature no popular presidential elections. In semi-presidential regimes, the president is head of state but not head of the government. In presidential regimes, the president is both, head of state and head of government. Only Ireland changed its democratic regime type from parliamentary to semi-presidential when adopting a new constitution in 1937.

^5^The sample includes elections from Austria, Belgium, Czechoslovakia, Denmark, Estonia, Finland, France, Germany, Greece, Iceland, Ireland, Italy, Latvia, Lithuania, Luxembourg, Netherlands, Norway, Poland, Portugal, San Marino, Spain, Sweden, Switzerland, United Kingdom, Yugoslavia, Argentina, Chile, Colombia, Cuba, Uruguay, United States of America, Canada, Australia, and New Zealand.

^6^We omit 14 elections in 1919 from the graph.

^7^The effective number of parties measure summarizes the number of parties after an election by weighting each parties influence by its size. The weights in the electoral parties measure are vote shares, while the weights in the parliamentary party index are seat shares: $1/\sum_{i}^{n}s_{i}^{2}$ , where *s* captures vote or seat shares and *n* is the number of parties.

^8^These are Latvia, Estonia, Yugoslavia, and Germany. Czechoslovakia is the fifth country that we classify as having survived until the German occupation began.

## References

[1] Golder M : Democratic Electoral Systems around the world, 1946–2000. *Elect Stud.* 2005;24(1):103–121. 10.1016/j.electstud.2004.02.008

[2] Bormann NC Golder M : Democratic Electoral Systems around the world: 1946-2011. *Elect Stud.* 2013;32(2):360–9. 10.1016/j.electstud.2013.01.005

[3] Bormann NC Golder NC : Democratic Electoral Systems around the world, 1946-2020. *Elect Stud.* 2022;78(3): 102487. 10.1016/j.electstud.2022.102487

[4] Tichelbaecker T Gidron N Horne W : What do we measure when we measure affective polarization across countries? *Public Opin Q.* 2023;87(3):803–815. 10.1093/poq/nfad033

[5] Stegmaier M Linek L Marcinkiewicz K : Heterogeneity in the effects of resources, proximity, and identity on preference voting in PR systems. *East Eur Polit.* 2024;40(2):239–255. 10.1080/21599165.2023.2244885

[6] Bol D Hunter A Fernandez GA : The psychological partisan effect of electoral systems: How ideology correlates with strategic voting. *Party Politics.* 2023;30(4). 10.1177/13540688231176975

[7] De Francesco F Tosun J : The enactment of public participation in rulemaking: A comparative analysis. *Swiss Polit Sci Rev.* 2023;29(1):21–36. 10.1111/spsr.12550

[8] Clark WR Golder M : Rehabilitating duverger’s theory: testing the mechanical and strategic modifying effects of electoral laws. *Comp Polit Stud.* 2006;39(6):679–708. 10.1177/0010414005278420

[9] Lupu N : Party brands in crisis: partisanship, brand dilution, and the breakdown of political parties in Latin America.Cambridge University Press: New York, NY,2016. Reference Source

[10] Choi S Furceri D Yoon C : Policy uncertainty and foreign direct investment. *Rev Int Econ.* 2021;29(2):195–227. 10.2139/ssrn.3752479

[11] Bormann NC : Uncertainty, cleavages, and ethnic coalitions. *J Polit.* 2019;81(2):471–486. 10.1086/701633

[12] Ochieng’ Opalo K : Legislative development in Africa: politics and postcolonial legacies.Cambridge University Press: New York, NY,2019. 10.1017/9781108684651

[13] Spoon JJ Klüver H : Responding to far right challengers: Does accommodation pay off?In: *Domestic Contestation of the European Union.*Routledge,2021;113–131. Reference Source

[14] Cornell A Møller J Skaaning SE : The real lessons of the interwar years. *J Democr.* 2017;28(3):14–28. 10.1353/jod.2017.0040

[15] Valentim V Dinas E : Does party-system fragmentation affect the quality of democracy? *Br J Polit Sci.* Forthcoming.

[16] Kaftan L Bormann NC : Polarization, fragmentation, and democratic deconsolidation in interwar europe. *Paper Presented at the Annual Meeting of the American Political Science Association.*August 31st to September 3rd. 2023 in Los Angeles, CA,2023.

[17] Boix C : Setting the rules of the game: The choice of electoral systems in advanced democracies. *Am Polit Sci Rev.* 1999;93(3):609–624. 10.2307/2585577

[18] Bormann NC Kaftan L : Replication Data for: Introducing the Democratic Electoral Systems data, 1919–1945. [Dataset], Harvard Dataverse, v1,2023. 10.7910/DVN/FWLXHM PMC11325140 39148582

[19] Mackie TT Rose R : The international almanac of electoral history.Macmillan: Basingstoke, UK, 3rd edition,1991.

[20] Nohlen D : Elections in the Americas - A Data Handbook Volume 1: North America, Central America, and the Caribbean.Oxford University Press: Oxford, UK,2005. Reference Source

[21] Nohlen D : Elections in the Americas - A Data Handbook Volume 2: South America.Oxford University Press: Oxford, UK,2005. Reference Source

[22] Nohlen D Stöver P : Elections in Europe.Nomos: Baden-Baden, DE,2010. 10.5771/9783845223414

[23] Boix C Miller M Rosato S : A complete Data Set of political regimes, 1800-2007. *Comp Polit Stud.* 2012;46(12):1523–1554. 10.1177/0010414012463905

[24] Przeworski A Alvarez ME Cheibub JA : Democracy and development: political institutions and well-being in the world, 1950–1990.Cambridge University Press, Cambridge, MA,2000. Reference Source

[25] Cheibub JA Gandhi J Vreeland JR : Democracy and dictatorship revisited. *Public Choice.* 2010;143(1):67–101. 10.1007/s11127-009-9491-2

[26] Marshall MG Gurr TR : Polity5 - political regime characteristics and transitions, 1800–2018.Technical report, Center for Systemic Peace,2020. Reference Source

[27] Coppedge M Gerring J Knutsen CH : V-dem country-year dataset v13. *Varieties of Democracy (V-Dem) Project.* 2023. 10.23696/vdemds23

[28] Singer JD Small M : Correlates of war project: international and civil war data, 1816–1992.Inter-University Consortium for Political and Social Research Ann Arbor, Michigan,1994. 10.3886/ICPSR09905.v1

[29] Gleditsch KS Ward MD : Interstate system membership: A revised list of the independent states since 1816. *Int Interact.* 1999;25:393–413.

[30] Cheibub JA : Presidentialism, parliamentarism, and democracy.Cambridge University Press,2007. Reference Source

[31] Ziegler DJ : Prelude to democracy: A study of proportional representation and the heritage of weimar Germany, 1871–1920. *Papers from the University Studies series (The University of Nebraska).* 1958;67. Reference Source

[32] Gamboa R Morales M : Deciding on the electoral system: chile’s adoption of proportional representation in 1925. *Lat Am Politics Soc.* 2015;57(2):41–66. 10.1111/j.1548-2456.2015.00269.x

[33] Duverger M : Political parties.Methuen, London,1963.

[34] Cox G : Making votes count: strategic coordination in the world’s electoral systems.Cambridge University Press, New York,1997. Reference Source

[35] Golder M : Presidential coattails and legislative fragmentation. *Am J Political Sci.* 2006;50(1):34–48. 10.1111/j.1540-5907.2006.00168.x

[36] Zeileis A Fisher JC Hornik K : colorspace: A toolbox for manipulating and assessing colors and palettes. *J Stat Softw.* 2020;96(1):1–49. 10.18637/jss.v096.i01

[37] Schvitz G Girardin L Rüegger S : Mapping the international system, 1886-2019: the cshapes 2.0 dataset. *J Conflict Resol.* 2022;66(1):144–161. 10.1177/00220027211013563

[38] Wickham H : ggplot2. *Wiley Interdiscip Rev Comput Stat.* 2011;3(2):180–185. 10.1002/wics.147

