# Supplementary material for: Introducing the Democratic Electoral Systems data, 1919-1945
Source: Open Res Eur. 2024 Sep 6;4:73. Originally published 2024 Apr 18. [Version 2] doi: 10.12688/openreseurope.17264.2 (PMC11325140; doi:10.12688/openreseurope.17264.2)
Supplement: Peer review history

# Reviewer report: Reviewer response for version 2

**DOI:** 10.21956/openreseurope.19960.r44003

**Published:** 2024-09-25

**Authors:** Pedro Riera

**Affiliations:** 1 University Carlos III of Madrid, Madrid, Spain

The authors have properly addressed all my main concerns and the data note can be accepted as it stands. I would like to thank and congratulate the authors on this nice and extremely useful addition to the state of the start. In my view, the dataset will be massively employed by experts in electoral systems all over the world.

Are sufficient details of methods and materials provided to allow replication by others?

Yes

Is the rationale for creating the dataset(s) clearly described?

Yes

Are the datasets clearly presented in a useable and accessible format?

Yes

Are the protocols appropriate and is the work technically sound?

Yes

Reviewer Expertise:

Comparative politics, electoral systems, voting behaviour.

I confirm that I have read this submission and believe that I have an appropriate level of expertise to confirm that it is of an acceptable scientific standard.

# Reviewer report: Reviewer response for version 1

**DOI:** 10.21956/openreseurope.18659.r40564

**Published:** 2024-08-14

**Authors:** Daniela Pastarmadzhieva

**Affiliations:** 1 University of Plovdiv "Paisii Hilendarski", Plovdiv, Bulgaria

The article „Introducing the Democratic Electoral Systems data, 1919-1945“, presents the most recent addition to the Democratic Electoral Systems around the world data. As the authors pointed out it encompasses the period from 1919 to 1945 and consists of 243 legislative lower house and presidential elections in 34 interwar democracies. The structure of the article is standard, consisting of an introduction, a description of the materials and methods, a substantial part, and a conclusion. The introduction is well structured and determines the framework and focus of the article. The methodology is described step-by-step, it is relevant for the goal of the study and is reproducible. The inclusion of regime types along with the electoral systems (and their sub-types) provides more options for analysis. The application of different conceptual frameworks to define democracy is another benefit for the users.

 Substantially, the new addition to the DES dataset has a wide range of benefits for scholars and for all interested in the elections. The historical background is rather significant in the explanation of the contemporary state in a given national context, tracing the origins of transformations. Of particular importance is the collection of information for a historical period, which enables researchers to expand the range of analyses. Thanks to the addition to the database, it is now possible to compare the period before and after the Second World War, institutional transformations (in particular electoral legislation), and changes in societies. It is possible to examine the dynamics in a country’s electoral rules from 1919 to the present by relating them to the dynamics in society. Alongside, associations between the structure of parliaments determined by the electoral rules and the decisions of states regarding the Second World War events might be identified. The above-mentioned are only part of the huge potential that the new database provides. In summary, current challenges cannot be fully understood without knowledge of the historical context, and DES 1919-1945 provides this opportunity to have accessible data on processes regarding the elections in contemporary democracies.

 The submitted article can be indexed as is, both structurally and substantively. However, concerning the DES, I would like to make a suggestion. Some of the countries that do not appear in the database are democracies nowadays. It seems to me that following the evolution of their electoral rules would contribute to a better understanding of the processes of democratization. Thus, if DES can be supplemented with more European countries although if not defined as democratic in the examined period, it will have a greater contribution to the comparative politics.

Are sufficient details of methods and materials provided to allow replication by others?

Yes

Is the rationale for creating the dataset(s) clearly described?

Yes

Are the datasets clearly presented in a useable and accessible format?

Yes

Are the protocols appropriate and is the work technically sound?

Yes

Reviewer Expertise:

Elections and electoral systems, comparative politics, political culture

I confirm that I have read this submission and believe that I have an appropriate level of expertise to confirm that it is of an acceptable scientific standard.

# Response

**Published:** 2024-08-26

**Authors:** Nils-Christian Bormann

*Dr. Daniela Pastarmadzhieva suggested to extend the DES 1919-1945 to countries which are democracies today but have not been democracies during the interwar period.*

Response: We agree with Dr. Daniela Pastarmadzhieva that this would be interesting and relevant. Unfortunately, we do not have the resources and time to extend the DES as of now but will keep this suggestion in mind for future extensions.

# Reviewer report: Reviewer response for version 1

**DOI:** 10.21956/openreseurope.18659.r40572

**Published:** 2024-06-10

**Authors:** Pedro Riera

**Affiliations:** 1 University Carlos III of Madrid, Madrid, Spain

This is a much-needed article (dataset) on electoral systems in democratic countries in the interwar period. The authors have managed to compile an immense quantity of reliable and useful data that I expect to be widely employed by comparativists of all over the world in the coming years. For this main reason, I support indexing of the article. Having said that, I am listing in the next paragraphs a battery of ideas that, in my view, could improve the dataset and the resulting article.

1. My main concern about the article is that electoral systems per se should play a bigger role in it. Let me offer a couple of examples:
  1. Figure 1 displays information on regime types across the world. However, a similar graph with information on electoral system in use in each country does not exist. Electoral systems are the main object of the article and, as such, they should occupy a more prominent position when offering descriptive information.
  2. The article starts by very briefly explaining the main elements of the new dataset (i.e., electoral rules, consequences of electoral rules, democracy indices and ID variables). However, I was missing some extra focus on the electoral rules variables. Which ones are included in the article? One can imagine that these are going to be the same that appear in the “sequel”, the widely acclaimed Bormann et al.’s “Democratic Electoral Systems around the world, 1946-2020” dataset, but this needs to be explicitly said. On top of that, I have always considered the omission of an “electoral thresholds” variable in the original dataset as one its main mistakes. The reader wonders if this is also the case here and, if so, why the authors have not included information on this important element of democratic electoral systems.
2. Elections as unit of analysis. In the previous *Electoral Studies* articles, the authors use as unit of analysis the election rather than the country. This sometimes can result misleading because the number of elections a country holds depends, among others, on the constitutional inter-election period. In order to be sure that the latter is not entirely responsible for this effect, I suggest that the authors include a new variable in the dataset that captures the duration of the constitutional inter-election period.
3. Dubious theoretical claims:
  1. In page 5, the article argues that the steeper decline of the number of elections in PR countries is due to the geographical clustering of these countries in Europe rather than the type of system of government in place in these countries. However, the reader wonders how the geographical clustering of parliamentary systems in Europe can lead to this result. What is the (theoretical) mechanism linking these two variables (that is, geography and democratic survival)? I am aware of several ideas in this regard, the existence of domino/diffusion effects being the most prominent ones, but these points need to be made clearer in the article.
  2. In page 9, when talking about the effects of formulas in presidential elections, the authors allude to the “relatively young age of many democracies” to justify the existence of null effects in this regard. However, these effects do exist when examining the role of formulas in legislative elections (see previous pages). How can it be this discrepancy whether democracies are of relatively the same age?
4. Small points (page 6):
  1. When talking about how uncommon mixed systems were in the interwar period, provide examples (for example, Denmark).
  2. Why not including a figure about frequency of types of PR electoral formulas?

Are sufficient details of methods and materials provided to allow replication by others?

Yes

Is the rationale for creating the dataset(s) clearly described?

Yes

Are the datasets clearly presented in a useable and accessible format?

Yes

Are the protocols appropriate and is the work technically sound?

Yes

Reviewer Expertise:

Comparative politics, electoral systems, voting behaviour.

I confirm that I have read this submission and believe that I have an appropriate level of expertise to confirm that it is of an acceptable scientific standard, however I have significant reservations, as outlined above.

# Response

**Published:** 2024-08-26

**Authors:** Nils-Christian Bormann

*Pedro Riera’s main concern has been that electoral systems per se play a minor role in the description of the dataset.*

Response: We appreciate this feedback. In addition to the Figures and descriptive analyses we had already included in the first version of the manuscript, we have now added Figure 4 and a corresponding description, providing more details about the distribution of electoral systems across the world.

*Pedro Riera has also wandered whether the DES data 1919-1945 contains information on the electoral thresholds, and if not, why this is the case.*

Response: The DES 1919-1945 data does not contain information on electoral thresholds because it went beyond the resources of this research project. Consider the following historical and contemporary examples: In Romania, ethnic minority representatives have a lower threshold to reach than their ethnic majority colleagues. In Poland, the threshold is lower for parties than for alliances of parties. In New Zealand and Germany, the electoral threshold only applies to parties that do not win a seat directly. Unlike in countries where thresholds are typically based on the percentage of votes a party gained, Slovenia bases its threshold on the number of seats won. In interwar Czechoslovakia, the threshold was based the absolute number of votes won, not the percentage – only determining whether a party could participate in distribution of votes on the second tier. Thus, deciding which electoral threshold to code and how to deal with many exceptional cases would have taken considerable time. We therefore kept the focus on the original DES coding instructions.

*Pedro Riera suggested to include information on the constitutional inter-election periods.* Response: As collecting data on on inter-election periods was fairly straight forward, we added two new variables to the DES 1919-1945: one indicates the length of the term for presidents and parliaments (in years), respectively, the other indicates the intervals in which elections are held (in years) in case parliaments are only renewed partially. For example, Argentina’s members of parliament were elected for four years, but elections took place every two years for half of the parliament.

*Pedro Riera furthermore raised some concerns regarding two “dubious theoretical claims”: The first is about the effect of the geographical clustering of parliamentary systems in Europe on their survival, and the second refers to the non-existing difference between the number of candidates running for presidential elections in plurality vs. absolute majority systems between 1919 and 1945.*

Response: We want to thank Pedro Riera for carefully reading our manuscript. We deleted the first theoretical claim, mostly because this note is supposed to present the dataset, and not to defend novel theoretical arguments. We now claim that the discrepancy between the interwar and post-World War II periods “raises important theoretical questions regarding the stability of presidential versus parliamentary systems.” Regarding the second claim: We added that this is a claim about presidential elections. The p residential and semi-presidential regimes in our sample are younger on average than parliamentary ones. For example, parliamentary Denmark, France, Norway, Switzerland, the UK had been democratic for more than ten years when our data starts recording elections in 1919. Among presidential democracies, only the US had been democratic for more than ten years.

*Pedro Riera furthermore suggested to provide examples, primarily when speaking about how uncommon mixed systems were in the interwar period.*

Response: We added France and Iceland as examples in the text. Denmark only ran one mixed election in 1918, a year that does not fall into our sampling period.

*Finally, Pedro Riera wondered why we did not include a figure about the frequency of PR electoral formulas.*

Response: The main reason is that such a graph is hard to read. There are 19 different PR electoral formulas, several of them with very few elections. We find it more informative to describe the frequency of the main formulas in the text.

# Reviewer report: Reviewer response for version 1

**DOI:** 10.21956/openreseurope.18659.r40566

**Published:** 2024-05-31

**Authors:** Düzgün Arslantas

**Affiliations:** 1 Cologne Center for Comparative Politics,, University of Cologne, Koln, Germany

The Democratic Electoral Systems (DES) dataset is an important addition to the existing scholarship on the electoral systems. The dataset includes a wide-range of information on 243 legislative lower house and presidential elections in 34 interwar democracies. The merit of the dataset stems from several factors. First, the paper covers a highly unique period in world history, i.e., the interwar years. Not only did this era witness the emergence and the spread of the most destructive political ideologies historically, but it also manifested the fragility of the so-called “established democracies” under non-democratic parties and leaders – even if they assumed power for a short period of time. Second, the dataset provides valuable insights into the trajectory of global electoral system changes, forming a foundational basis for future electoral engineering practices. Third, the dataset bridges a wide range of datasets through multiple identification variables. This allows researchers to make comparisons across different datasets and select the most suitable dataset for strengthening the empirical analysis. Fourth, the interdisciplinary nature of the DES dataset allows for the verification of proposed arguments against other aspects of electoral politics.

 Despite these merits, some deficiencies should be stated. To begin with, first-hand sources are crucial for historical analysis, as few scholars possess sufficient resources to re-evaluate this period. This means that most scholars will possibly adopt the findings of this research without detailed investigation. From this perspective, the fact that some of the languages of the countries analyzed are not spoken by the research assistants may limit the reliability of the findings. Second, in which respect the DES dataset improves the understanding of the diffusion of electoral rules across Europe and the Americas is unclear. Historically, most of the colonized countries adopted the electoral systems of their imperial rulers. It remains to be seen whether such kind of diffusion can be mentioned for democracies as well. Third, additional information on how many elections are classified as “democratic” based on this reductionist account of the “democracy-dictatorship” classification would be beneficial. As widely noted, a binary classification overlooks the increasing prevalence of regimes that fall between these extremes. Finally, I am not fully convinced whether using the sub-title “democratic electoral systems” is conceptually appropriate. If we mention “democratic electoral systems,” then are there also “authoritarian” electoral systems or even “totalitarian” ones? How do they look like? Imagine an electoral authoritarian regime with little or no ethnic and religious pluralism. If this country adopts an electoral system that maximizes descriptive representation of the society, can we call the electoral system “democratic”?

Are sufficient details of methods and materials provided to allow replication by others?

Yes

Is the rationale for creating the dataset(s) clearly described?

Yes

Are the datasets clearly presented in a useable and accessible format?

Yes

Are the protocols appropriate and is the work technically sound?

Yes

Reviewer Expertise:

comparative politics

I confirm that I have read this submission and believe that I have an appropriate level of expertise to confirm that it is of an acceptable scientific standard.

# Response

**Published:** 2024-08-26

**Authors:** Nils-Christian Bormann

*First, Düzgün Arslantas has raised the concern that our research team did not speak all languages spoken in the countries included in the dataset, potentially introducing bias.* Response: We agree that this may be a problem, although it is a common in cross-national research. We would like to highlight that our research team was able to read source material in Dutch, English, Finnish, French, German, Italian, Serbo-Croatian, Spanish, and Swedish. Thus, we spoke the languages of 26 of the 34 countries included in the dataset. Moreover, we also relied on data compiled by others in a comparative manner such as Mackie and Rose (1991), Nohlen (2005), and Nohlen and Stöver (2010). There compilations are predominantly authored by country experts, who speak the language and know the cultural specificities of “their” country. We are confident that this allowed us to minimize bias as much as possible. We have clarified this point in the main text (see point 4 in the Section “Materials & Methods”).

*Second,* *Düzgün Arslantas pointed out that it remains to be seen whether the DES dataset can improve our understanding of the diffusion of electoral rules across Europe and the Americas.*

Response: We agree and apologize for the unprecise wording. We have clarified our statement. We now say: “Finally, the DES 1919-1945 release will prove a valuable resource for scholars who study the origins of electoral systems, for example to explore the role of diffusion of electoral rules across Europe and the Americas.”

*Third, Düzgün Arslantas asked for additional information on how many elections are classified as democratic, given the increasing prevalence of regimes that fall between the two extremes of authoritarian and democratic systems.*

Response: We apologize for not having been clear enough. We include all elections that have been classified as democratic by any of the following datasets: Boix, Miller, and Rosato (2012), the dichotomous Democracy and Dictatorship classification (DD), V-Dem, and Polity5. We did not include elections that took place in periods coded as undemocratic by all four datasets listed above. We have chosen this approach to allow researchers themselves to select elections that they deem to be “democratic enough” for their research interest. We have rewritten the first paragraph of the Section “Democratic Electoral Systems, 1919-1949” to clarify our approach.

*Forth, Düzgün Arslantas mentions conceptual issues with the term “democratic electoral system” since this suggests that there are also undemocratic elections.*

Response: We carefully disagree here but believe that this disagreement is rooted in a misunderstanding of our position due to a lack of clarity in the previous version of the manuscript. There are indeed elections taking place in non-democratic systems (e.g., Russia as of today), and there is a large body of literature surrounding these elections (see for example Gandhi and Lust-Okar, 2009). But of course, we do not claim that political systems such as Russia are democracies only because they have elections. This is why we fall back on the four datasets listed above to define our universe of cases: elections that happen in a (at least minimally) democratic environment. We ignore elections that take place in non-democratic systems.
